# Multiple amygdaloid divisions of arcopallium send convergent projections to the nucleus accumbens and neighboring subpallial amygdala regions in the domestic chicken: a selective pathway tracing and reconstruction study

**DOI:** 10.1007/s00429-016-1219-8

**Published:** 2016-04-06

**Authors:** János Hanics, Gyöngyi Teleki, Alán Alpár, Andrea D. Székely, András Csillag

**Affiliations:** 1 Department of Anatomy, Histology and Embryology, Faculty of Medicine, Semmelweis University, 58. Tuzolto utca, 1094 Budapest, Hungary; 2 MTA-SE NAP B Research Group of Experimental Neuroanatomy and Developmental Biology, Hungarian Academy of Sciences, Budapest, Hungary

**Keywords:** Viscerolimbic, Subpallium, Basal ganglia, Avian, Calcium binding proteins, Dopamine

## Abstract

Retrograde tracing with choleratoxin B, injected into the nucleus accumbens (Ac) and bed nucleus of stria terminalis, lateral part (BSTL), yielded labeled perikarya in a ring-shaped area of arcopallium, including dorsal and hilar subdivisions, with a wedge-shaped node of dense accumulation in the amygdalopiriform area (APir). Also, the position of source neurons for this arcopallio-subpallial pathway was verified by anterograde tracing. Three subregions of arcopallium (amygdalopiriform, dorsal, hilar) were injected with dextran (10 kDa), and fibers and terminal fields were detected in Ac, BSTL and extended amygdala (EA). Most abundant projections to Ac arose from APir. The study enabled precise description of the main output fiber streams: the dorsal stream follows the dorsal border of arcopallium and, continuing in the ventral amygdalofugal tract, it traverses the EA and the BSTL before reaching the Ac. The ventral stream of fibers enters the EA along the ventral subpallial border and terminates in the basal nucleus and ventral pallidum. The course of the pathway was reconstructed in 3D. Retrogradely labeled arcopallial neurons were devoid of DARPP-32. DARPP-32 was present in the Ac but not the BSTL. No colocalization between the calcium binding proteins calbindin, parvalbumin and calretinin, and retrogradely labeled neurons was detected, despite a considerable territorial overlap. This finding further supports the excitatory nature of the arcopallial-accumbens pathway. Conjoint and convergent amygdalar input to EA, including BSTL, as well as to Ac subregions likely transmits fear and aggression related signals to both viscerolimbic (EA) and learned reward- and motivation-related (Ac) ventrobasal forebrain regions.

**Electronic supplementary material:**

The online version of this article (doi:10.1007/s00429-016-1219-8) contains supplementary material, which is available to authorized users.

## Introduction

The ventrobasal forebrain nuclei, including the nucleus accumbens (Ac), bed nucleus of stria terminalis, lateral part (BSTL) and other components of extended amygdala (EA), ventral pallidum (VP) and cholinergic cell groups (such as the basal nucleus of Meynert) have been implicated in the initiation and reinforcement of movements, motivation and emotion, reward and aversion (Alheid et al. 1995; Alheid and Heimer 1988; de Olmos et al. 2004; Li and Sakaguchi 1997). These regions are extensively connected with the amygdala, whose involvement in emotional responses is also well established (Phelps and LeDoux 2005; Swanson 2000). While the majority of relevant studies focused on mammalian species, our laboratory has been active in revealing a similar role of relevant systems in birds, in which the anatomical structures and connectivity of ventrobasal forebrain nuclei show extensive homologies with their mammalian counterparts (Csillag 1999; Csillag et al. 1997, 2008; Csillag and Montagnese 2005; Jarvis et al. 2005; Kuenzel et al. 2011; Reiner et al. 2004). As a model system, young domestic chicks offer a unique opportunity for studying, learning and motivation because of their early maturation (precocial development of a nidifugous species). For example, one-trial passive avoidance training is a simple and reproducible way of investigating early adaptive learning processes (Rose 2000). It has been established that, of all telencephalic regions, the basal ganglia show the highest degree of homology between birds and mammals. This may serve as justification for a comparative approach in the investigation of neural mechanisms, such as motivation of elementary actions, which have been conserved throughout vertebral evolution in both mammals and Sauropsida (diverging over 200 million years ago).

Earlier results from our laboratory, based on electron microscopic immunocytochemistry, have indicated the presence of l-Asp and l-Glu in excitatory axodendritic boutons in the striatum/accumbens region of rats and chickens (Adam and Csillag 2006; Hanics et al. 2012). The source of these terminals proved to be the basolateral amygdala (BLA) in the rat, whereas in chicks the source region was located in the amygdaloid arcopallium (Hanics et al. 2012). Further, to investigate the specificity of l-Asp containing input pathways in the domestic chicken, it was necessary to carry out a detailed pathway tracing study, combined with immunohistochemistry, relevant to the chemical nature of source neurons and of potential target areas.

Description of projection patterns between select brain areas critically relies on domain-specific tract tracing approach. To project the arcopallial output onto the avian ventrobasal forebrain regions including the Ac, EA, BSTL, we applied high precision region-specific in vivo anterograde and retrograde tracing experiments, including dual tracing by simultaneously using different dyes. The analysis of serial high power magnification multi-tile section reconstructions allowed us to map a hitherto uncharacterized amygdalosubpallial pathway.

## Materials and methods

### Animals

Thirty 7–14 days old *Hunnia* broiler hybrid domestic chickens (*Gallus domesticus*) were used. Food and water were available ad libitum. Experimental procedures on birds, including stereotaxic injections and transcardial perfusion were approved by the Semmelweis University and conformed to the European Convention for the Protection of Vertebrate Animals used for experimental and other scientific purposes (Protocols: ETS No. 170, ETS No.123).

### Tracing studies

Animals were anesthetized intramuscularly with a mixture of Ketamine (50 mg/kg b.wt.) and xylazine (4 mg/kg b.wt.) and placed in a Kopf stereotaxic instrument, maintained at 39 °C (Supertech heat pad) during surgery. The beak bar was set at −10 mm below horizontal. The skull was exposed by a skin incision, and small holes at corresponding coordinates were drilled through the skull to access the brain. Tracers were injected stereotaxically using a 1.0 µL Hamilton syringe mounted on a Kopf microinjector unit. Retrograde or anterograde tracers (0.04 µL in volume) were deposited into the corresponding brain region by slow pressure injection lasting for 5 min. The needle was retracted only after a 15 min resting interval to avoid leakage along the injection canal. Coordinates of targeted brain regions were previously verified in pilot experiments by ink injections (methylene blue) and based on the brain atlas of Puelles et al. (2007) for a more refined subregional analysis, and then compared with and projected onto the brain templates by Kuenzel and Masson (1988). It has to be noted that the anteroposterior (AP) coordinates described here are not an exact match of those demonstrated in the Puelles atlas due to a slight difference between the head angles (the cited author specifies a declination value, whereas, in our case, the position of beak bar is specified and set in the stereotaxic frame in mm below interaural line).

### Retrograde and anterograde tracer injections

Animals received unilateral dual or single injections of two different retrograde tracers, Alexa Fluor^®^ 488 (or 594) conjugated choleratoxin B subunit (CTb; Molecular Probes, Eugene, OR; 1 %, dissolved in PBS) and Fast Blue (FB, Polysciences, Warrington, PA; 5 %, dissolved in distilled water) into the ventrobasal forebrain. In dual retrograde tracing experiments, CTb was injected first into the BSTL-Ac at the stereotaxic coordinates anteroposterior (from bregma, also coinciding with interaural): +4.30 to 4.40 mm, lateral 0.79–0.82 mm, dorsoventral –5.57 to 6.07 mm which was followed by the administration of FB after 20 min 0.5 mm further lateral to the previous injection coordinates (medial striatum—MSt). Notably, the AP value specified above, corresponds approximately to the telencephalic topography depicted at interaural 5.2 mm in the brain atlas of Puelles et al. (2007), see the remark above. According to the atlas of Kuenzel and Masson (1988), this AP coordinate corresponds approximately to the value of *A* 9.4 mm. For the injections in the same animal separate, Hamilton syringes were used. Single CTb injections were applied at the BSTL-Ac coordinates.

Anterograde tracer injection was carried out by using Alexa Fluor^®^ 594 conjugated dextran 10 kDa (D594, Molecular Probes, Eugene, OR; 10 %, dissolved in distilled water). Animals received injection of D594 into the dorsolateral (APir coordinates anteroposterior +1.50 mm, lateral −6.74 mm, dorsoventral −3.55 mm), dorsal (ADo coordinates anteroposterior +1.50 mm, lateral −5.50 mm, dorsoventral −5.00 mm) and hilar (AHil coordinates anteroposterior +1.50 mm, lateral −4.60 mm, dorsoventral −6.00 mm) parts of the arcopallium using an identical Hamilton syringe. Notably, the AP value specified above, corresponds approximately to the telencephalic topography depicted at interaural 1.84 mm in the brain atlas of Puelles et al. (2007), see the remark above. According to the atlas of Kuenzel and Masson (1988), this AP coordinate corresponds approximately to the value of *A* 6.4 mm. Therefore, to make orientation easier for the avian neuroanatomists’ community, we display the coordinates of the latter, better-known atlas in our figures throughout. Animals were allowed to recover after surgery, and had access to food and water ad libitum.

### Perfusion and sectioning

On fourth and seventh day after retrograde and anterograde tracing, respectively, animals were deeply anesthetized intramuscularly with a mixture of ketamine (50 mg/kg b wt) and xylazine (4 mg/kg b wt), and transcardially perfused first with 50 mL physiological saline (0.9 % NaCl) followed by 250–300 mL 4 % paraformaldehyde in 0.1 M phosphate buffer (PB, pH 7.4). The brains were removed from the skull, postfixed at 4 °C in 4 % paraformaldehyde in PB overnight, and subsequently, transferred to 30 % sucrose (diluted in PB, 4 °C, for 2 days) for cryoprotection. Brains were sectioned at 70 μm on a Leica freezing microtome in the coronal plane. Sections were stored at 4 °C in 0.1 % sodium azide in PB until further processing.

### Tissue processing, immunochemistry, immunohistochemistry

We used retrograde and anterograde tracers that were directly labeled by fluorescent dyes. To investigate the labeled perikarya and axonal fibers, free-floating sections were rinsed in PB, mounted on gelatin-coated glass slides and coverslipped with glycerol–PBS (1:1) or Surgipath Micromount mounting medium (Leica Biosystems, Richmond, IL).

For multiple labeling experiments, free-floating sections were rinsed in PB (pH 7.4). Nonspecific immunoreactivity was suppressed by incubating our specimens in a cocktail of 5 % normal donkey serum (NDS; Jackson), and 0.3 % Triton X-100 (Sigma) in PB for 2 h at 22–24 °C. Sections were exposed (72 h at 4 °C) to select combinations of primary antibodies (Hemmings et al. 1987; Thomsen et al. 2010; Tomassy et al. 2014; Yamamoto et al. 2012) (Table 1) diluted in PB to which 0.1 % NDS and 0.3 % Triton X-100 had been added. After extensive rinsing in PB, immunoreactivities were revealed by species-specific carbocyanine (Cy) 2 or 5-tagged secondary antibodies raised in donkey [1:500 (Jackson), 24 h at 4 °C]. Glass mounted sections were coverslipped with Surgipath Micromount mounting medium.

**Table 1:** List of markers used for immunofluorescence labeling

Marker	Source	Host	IH dilution	References
DARPP-32	From H.C. Hemmings	Mouse, mc^a^	1:2000	Hemmings et al. (1987)
Calbindin D28k	Synaptic Systems	Guinea-pig, pc^b^	1:1000	Yamamoto et al. (2012)
Calretinin	Synaptic Systems	Guinea-pig, pc^b^	1:1000	Tomassy et al. (2014)
Parvalbumin	Sigma	Mouse, mc^a^	1:2000	Thomsen et al. (2010)

^a^Monoclonal antibody

^b^Polyclonal antibody

### Imaging and 3D reconstruction

Survey images were captured on an Olympus BX-51 epifluorescent microscope using 4×, 10×, 20× and 40× objectives (Plan-Apochromat 4×/0.2, 10×/0.45, 20×/0.8 or 40×/1.46), equipped with a digital camera, using the image capturing programs Viewfinder Lite and Studio Lite. Alternatively, overview images were taken on a 780LSM confocal laser scanning microscope (Zeiss) at 10× magnification and using the auto-tile-and-stitch function. Photomicrographs were compared with adequate brain atlas charts (Kuenzel and Masson 1988; Puelles et al. 2007) to define the position of labeled cells/axons. Sections processed for multiple immunofluorescence histochemistry were inspected and images acquired on the 780LSM confocal laser scanning microscope at 10×, 20× or 63× primary magnification (Plan-Apochromat 10×/0.45, 20×/0.8 or 63×/1.40), using minimal optical slice thickness (0.7–0.9 µm) at highest power imaging. Emission spectra for each dye were limited as follows: Cy2 (505–530 nm), Alexa Fluor 594 (560–610 nm), and Cy5 (650–720 nm). Multi-panel figures were assembled in CorelDraw X5 (Corel Corp.). Templates for schematic drawings of coronal brain sections were taken from http://www.avianbrain.org/atlases.html.

For high fidelity tract tracing, multiple middle-power magnification (100×) photos were taken that covered the unilateral basal telencephalic section. Photos were stitched using the Panorama option of IrfanView software. Altogether, 25 multi-tile images were assembled that included the total craniocaudal extension of the arcopallial-striatal tracts (coordinates 1.60–6.64 mm measured cranial to interaural line, based on the atlas of Puelles et al. (2007) and investigated for individual axons or axonal tracts. A corresponding series of D594/DARPP-32 double-labeled images were used to trace the interrelation between the arcopallial tract and BSTL in the ventrobasal forebrain for reconstruction. Three-dimensional modeling of the arcopallial tracts was based upon these two image series by using the Reconstruct software (Fiala 2005).

## Results

### The medial striatum is heterogeneous in its afferentation pattern

**Fig. 1:**
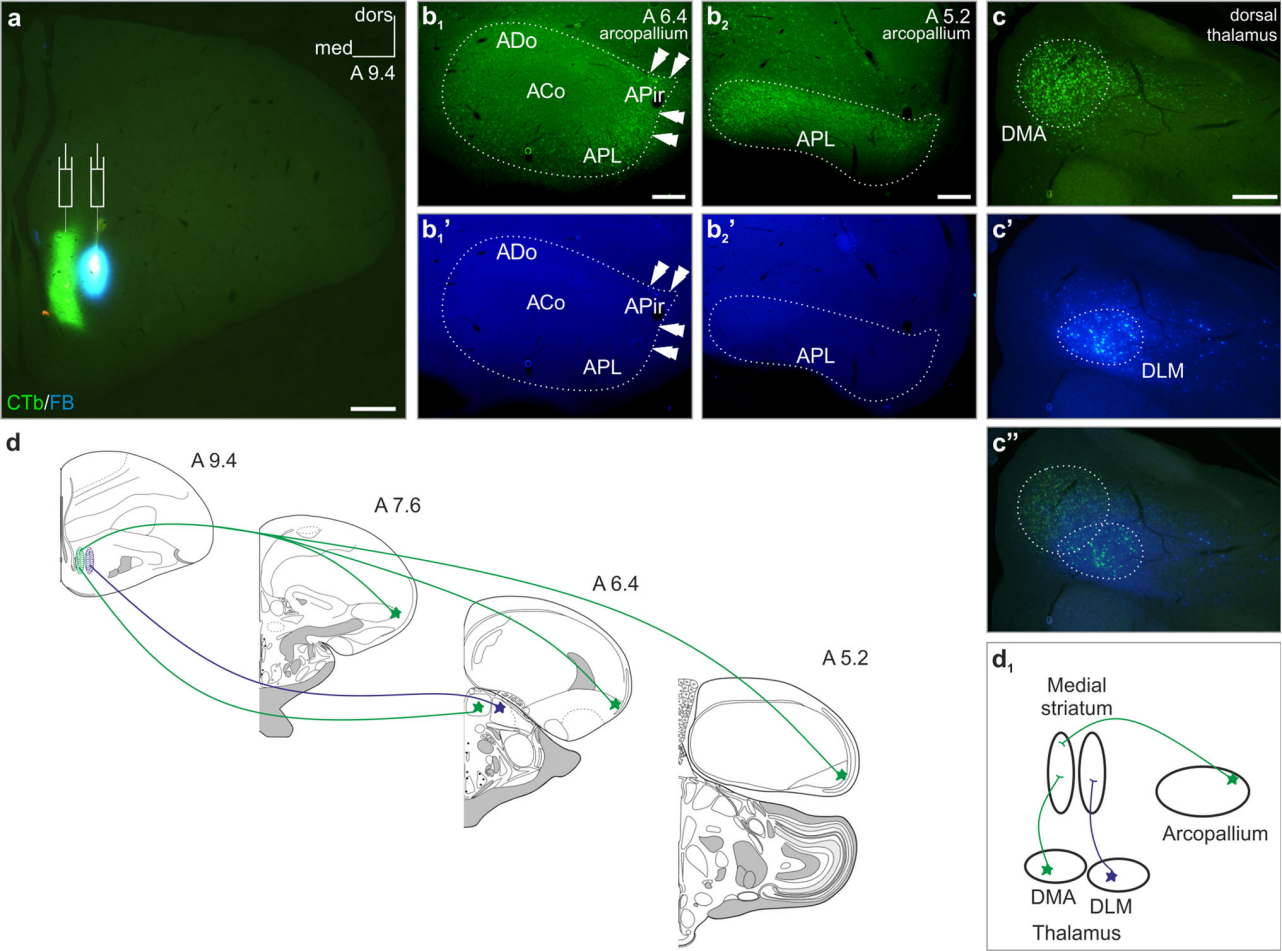
The medial striatum is inhomogeneous in its afferentation pattern. Simultaneous injections (appropriate *symbols* in **a**) of Alexa Fluor^®^ 488 conjugated choleratoxin B subunit (CTb) and Fast Blue (FB) retrograde tracers into the medial and lateral divisions of the medial striatum, respectively. **b**_**1**_, **b**_**2**_ CTb-labeled (CTb^+^) somata (*arrowheads*) were detected only in the peripheral part of arcopallium, including the dorsal and posterolateral part of the arcopallium (ADo and APL, respectively), with an outstanding density in its lateral region previously termed as the amygdalopiriform area (APir) (Puelles et al. 2007). **b**_**1**_ **′**, **b**_**2**_ **′** The arcopallium remained spared from FB-labeled (FB^+^) cell bodies. **c**–**c′′** The striatal projection of the dorsal thalamus showed a medial–lateral topology with a narrow overlap between these territories: CTb^+^ perikarya were restricted to the anterior dorsomedial nucleus (DMA), whilst FB^+^ cell bodies to the medial part of the dorsolateral anterior thalamic nucleus (DLM). **d**, **d**_**1**_ Schemata demonstrating the dual character of the retrogradely traced afferentation of the medial striatum (coronal section drawings were modified after the templates: http://www.avianbrain.org/nomen/Chicken_Atlas.html. *Numbers at the right upper corner* of the drawings and images indicate distance in millimeters AP according to Kuenzel and Masson (1988)). White dotted lines mark the outlines of arcopallium. *ACo* arcopallial core, *dors* dorsal, *med* medial. *Scale bars* 1 mm (**a**), 200 µm (**b**_**1**_, **b**_**2**_, **c**)

To identify the select arcopallial cell group (“amygdaloid arcopallium”) establishing an amygdalostriatal (amygdalosubpallial) pathway, we injected two different retrograde tracers, Alexa Fluor^®^ 488 conjugated choleratoxin B subunit (CTb) and Fast Blue (FB), into the medial and lateral divisions of the medial striatum of chicks, respectively (Fig. 1a). When mapping the arcopallium in its complete craniocaudal extension for retrogradely labeled CTb^+^ perikarya, we found a large number of neurons in its peripheral domain with an outstanding density in its dorsolateral part (Fig. 1b_1_), a region that continued caudally towards a seemingly more ventral position (Fig. 1b_2_). At the same time, we were unable to identify the FB^+^ cell bodies in any regions of the arcopallium (Fig. 1b_1_′, b_2_′). In the dorsal thalamus, CTb^+^ and FB^+^ neurons outlined a minimally overlapping region, with CTb^+^ perikarya populating the anterior dorsomedial nucleus (DMA) and FB^+^ perikarya the medial part of the dorsolateral anterior thalamic nucleus (DLM) (Fig. 1c–c′′). Thus, the simultaneous retrograde tracing from the medial and lateral parts of the medial striatum (MSt) allowed us to identify a different projection pattern from the arcopallium and thalamus (Fig. 1d, d_1_): while both adjacent tracer deposits led to markedly separate labeling sites in the dorsomedial thalamus, only one of them (the medial) yielded backfilled cells in the arcopallium. Discrete thalamic labeling could, thus serve as validation of the selective tracing method.

### The arcopalliofugal (amygdalofugal) tract terminates in a select domain of the ventrobasal forebrain

**Fig. 2:**
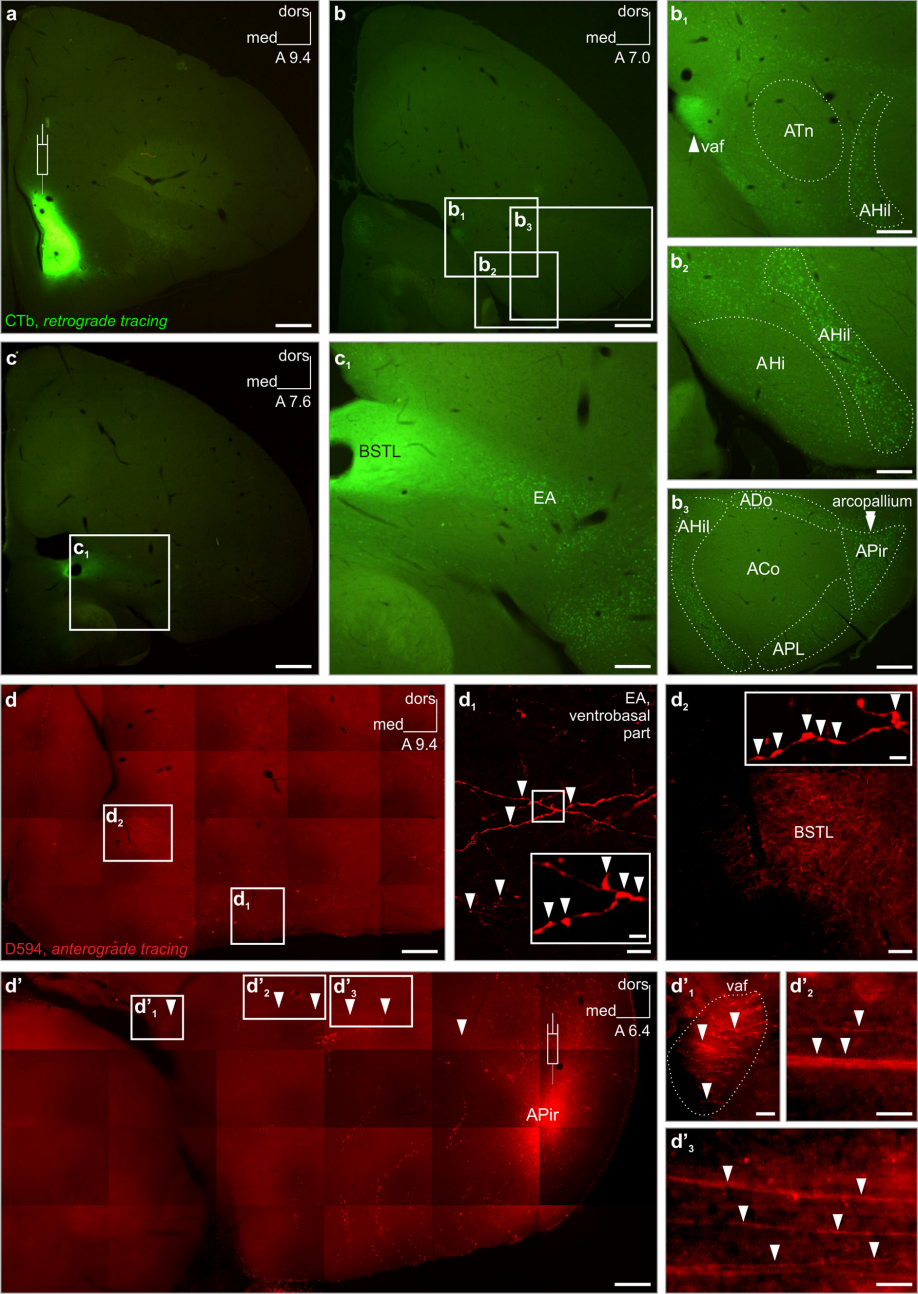
In vivo retrograde and anterograde tracing identify the exact pathway of an arcopallial tract which ends in a restricted subdivision of the ventrobasal forebrain. **a** Injection (appropriate *symbol* in **a**) of Alexa Fluor^®^ 488 conjugated choleratoxin B subunit (CTb) restricted to a medial juxtaventricular zone including the lateral part of the bed nucleus of stria terminalis (BSTL), nucleus accumbens (Ac) and a small part of medial striatum. Retrogradely labeled fibers can be traced throughout the ventral amygdalofugal pathway (vaf, *arrowhead* in **b**_**1**_). CTb-labeled somata were detected in several domains of the arcopallium at A7.0 **(b)**, including the hilar (AHil) (**b**_**1**_, **b**_**2**_, **b**_**3**_, *dotted outline*), dorsal (ADo), posterolateral (APL) and amygdalopiriform (APir) subunits that marked out a *ring-shaped* area (**b**_**3**_, *dotted outline*), with the highest density of labeled perikarya (*double arrowheads* in **b**_**3**_) in a wedge-shaped division (termed amygdalopiriform area by Puelles et al. (2007), APir) in the dorsolateral arcopallium. The amygdaloid taenia (ATn), the amygdalo-hippocampal area (AHi) and the core of the arcopallium (ACo) were typically spared from CTb-labeled cell bodies (**b**_**1**_–**b**_**3**_). **c**, **c**_**1**_ More cranially (**c**, **c**_**1**_), a large number of CTb^+^ neurons were identified which were dispersed throughout the extended amygdala (EA). High power multi-tile serial imaging (**d**–**d′**) allowed us to trace anterogradely labeled arcopallial fibers in coronal telencephalic brain sections. Injection (appropriate symbol in **d′**) of Alexa Fluor^®^ 594 conjugated high-molecular-weight (10 kDa) dextran (D594) into the APir filled latero-medially projecting axons (*arrowheads* in **d′**, **d**_**1**_ **′**, **d′**_**2**_, **d′**_**3**_) that passed subsequently through a region/pathway previously identified as vaf (*dotted outline* and *arrowhead* in **d′**_**1**_, compare also with **b**_**1**_). D594^+^ axons passed through and terminated in the BSTL and adjacent Ac (*arrowheads* in *inset* of **d**_**2**_ indicate terminal boutons in BSTL). Labeled axons were alternatively traced in the EA along the ventral subpallial border (**d**_**1**_, *arrowheads*). Cranio-caudal levels (**a**–**d′**) of coronal sections are indicated in millimeters AP according to Kuenzel and Masson (1988). *dors* dorsal, *med* medial. *Scale bars* 1 mm (**a**, **b**, **c**) 500 µm (**d**, **d′**), 250 µm (c_1_), 200 µm (**b**_**1**_–**b**_**3**_), 100 µm (**d**_**2**_), 10 µm (**d**_**1**_, **d′**_**2**_, **d′**_**3**_), 5 µm (**d′**_**1**_), 2 µm (*inset* in **d**_**1**_, **d**_**2**_)

To unequivocally prove and describe the exact projection pattern of the arcopallium onto the striatal region, we carried out correspondingly planned retrograde and anterograde tracing experiments (Fig. 2). Retrograde tracer (CTb) deposited into the Ac, including the juxtaventricular zone of the BSTL (Fig. 2a), labeled a large number of axons in the ventral amygdalofugal pathway (vaf) (Fig. 1b_1_) and perikarya in outstanding density in a dorsolateral wedge-shaped region of the arcopallium, termed as the amygdalopiriform area (APir) (Puelles et al. 2007) (Fig. 2b_3_). Albeit in lower densitites, CTb^+^ cell bodies were detected in the hilar (AHil), dorsal (ADo) and posterolateral (APL) parts of the arcopallium (Fig. 2b_2_, b_3_). No (or very few) labeled neurons were identified in the amygdalo-hippocampal (AHi), taenial (ATn) or core domains of the arcopallium at the same time (Fig. 2b_1_–b_3_). More cranially, retrogradely labeled neurons appeared in a large number throughout the extended amygdala (EA) (Fig. 2c, c_1_).

**Fig. 3:**
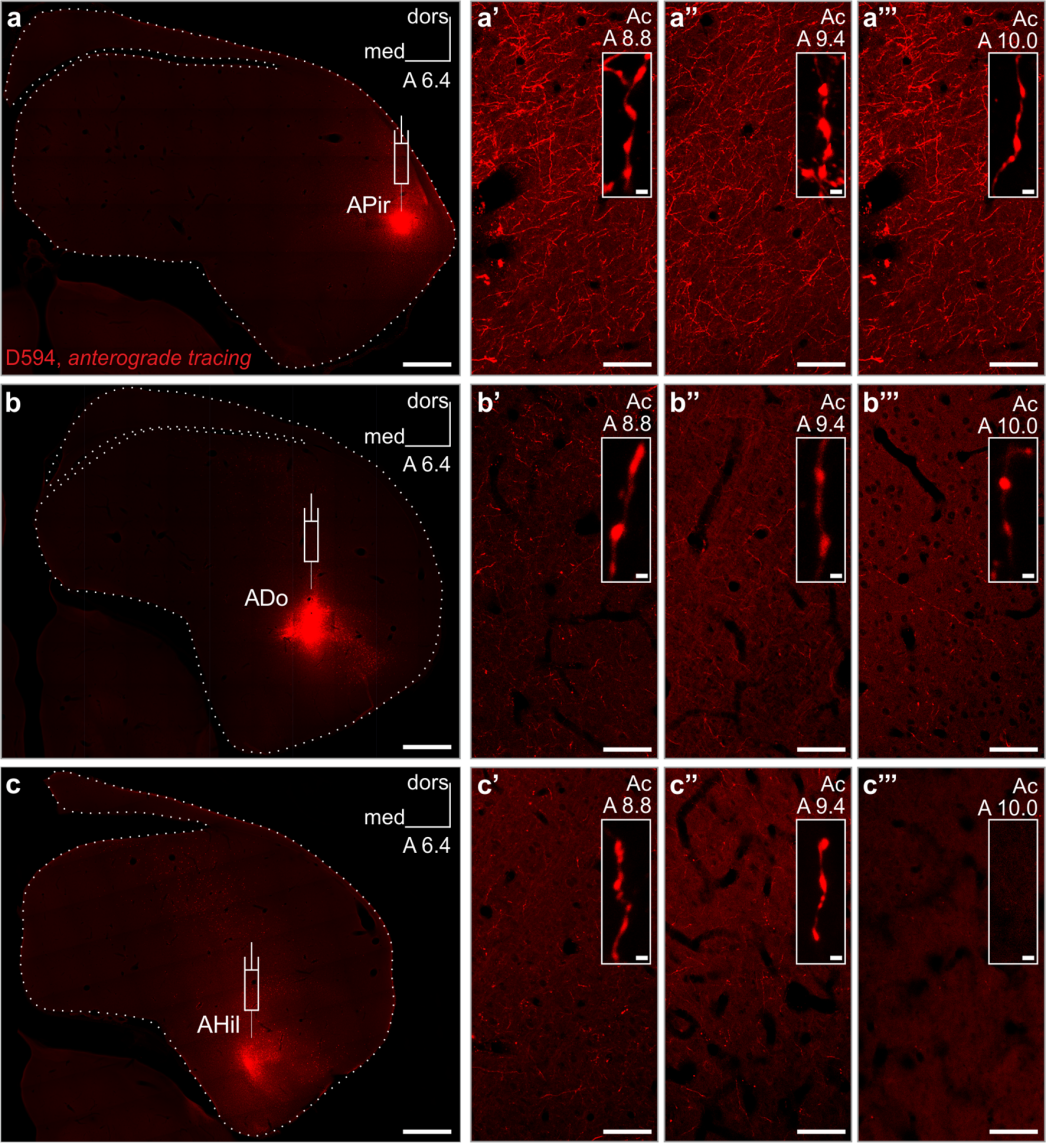
The arcopallium projects domain-specifically onto the nucleus accumbens (also representative of other ventrobasal forebrain projections). **a**–**a′′′** D594^+^ axons traced anterogradely from the amygdalopiriform area (APir) of the arcopallium, terminating in both the rostral, intermediate and caudal parts of the nucleus accumbens (Ac) in great density. Representative *insets* demonstrate varicose axons under high magnification. **b**–**b′′′** Injection placed into the dorsal arcopallium (ADo) labeled fewer terminals throughout the Ac. Representative *insets* demonstrate varicose axons under high magnification, though such axons were rather sporadic in the rostralmost Ac. **c–c′′′** The medial, hilar division of the arcopallium (AHil) gave rise to axons that terminated in the caudal and intermediate, but not the rostral, part of the Ac. Note the low density of terminal axons even in the intermediate and caudal parts of the Ac. Representative *insets* demonstrate varicose axons under high magnification. Here, due to an overall scarcity of varicose fibers, no such element could be indicated in the rostralmost Ac. **a**–**c** Appropriate symbols indicate the injection sites. Due to low intensity of section images (optimized for the fluorescent signal of tracer deposit), dotted lines indicate the outlines of sections. **a**–**c′′′** Cranio-caudal levels of coronal sections are indicated as distance in millimeters AP according to Kuenzel and Masson (1988). Abbreviations: D594 Alexa Fluor^®^ 594 conjugated high-molecular-weight (10 kDa) dextran, *dors* dorsal, *med* medial. *Scale bars*: 1 mm (**a**–**c**), 70 µm (**a′**–**a′′′**, **b′**–**b′′′**, **c′**–**c′′′**), 2 µm (*insets* in **a′**–**a′′′**, **b′**–**b′′′**, **c′**–**c′′′**)

Anterograde tracing using Alexa Fluor^®^ 594 conjugated high-molecular weight (10 kDa) dextran (D594) from the APir led to corresponding results (Fig. 2d, d_1_′, see also Fig. 3a–a′′′), verifying the presence of multiple axons arising from the APir in the vaf and BSTL. To identify the route of the investigated amygdalosubpallial pathway with high fidelity, we carried out total brain section scanning using high power magnification and subsequent multi-tile-stitching on complete craniocaudal series of the arcopallium (Fig. 2d–d′_3_, see also Electronic Supplementary Material). We found that axons arising from the APir follow two pathways: (1) medially along the dorsal border of the arcopallium (Fig. 2d′, d′_2_, d′_3_) to reach the vaf (Fig. 2d′_1_) with terminal endings subsequently identified in the BSTL (Fig. 2d, d_2_, also compare with Fig. 2a) or (2) a ventral course, passing through the ventrobasal part of EA (Fig. 2d, d_1_), and then invading the nucleus basalis and olfactory tubercle. The latter route was found typical for those fibers arising from more rostral levels of the arcopallium (Electronic Supplementary Material).

### Arcopallial domains show principal differences in their connectivity with the avian nucleus accumbens

Retrograde tracing from the Ac labeled perikarya in a dorsolateral wedge-shaped region of the arcopallium in outstanding density, whereas further—far less abundant—cell bodies were detected along the periphery of the arcopallium, leaving the “core” of the arcopallium spared from labeled neurons (Fig. 2b_3_). To investigate the exact projection pattern of the arcopallial “belt”, we injected the anterograde tracer D594 into its dorsolateral (APir), dorsal (ADo) and medial, hilar (AHil) divisions (Fig. 3a–c) and mapped the caudal, intermediate and rostral regions of the Ac for terminating axons (Fig. 3a′–a′′′, b′–b′′′, c′–c′′′). D594 injection led to a dense axonal and terminal labeling throughout the Ac when targeting the wedge-shaped APir (Fig. 3a–a′′′). In contrast, anterogradely labeled fibers within the Ac were identified in a dramatically smaller number when the injection sites were restricted to the ADo (Fig. 3b–b′′′) or AHil (Fig. 3c–c′′′) divisions of the arcopallium. Actually, no fibers were detected in the rostral most part of the Ac when D594 had been administered into the hilar region of the arcopallium (Fig. 3c′′′).

### Arcopallial fibers typically target the medial (DARPP-32^−^), also invading the lateral (DARPP-32^+^) division of the ventrobasal forebrain

**Fig. 4:**
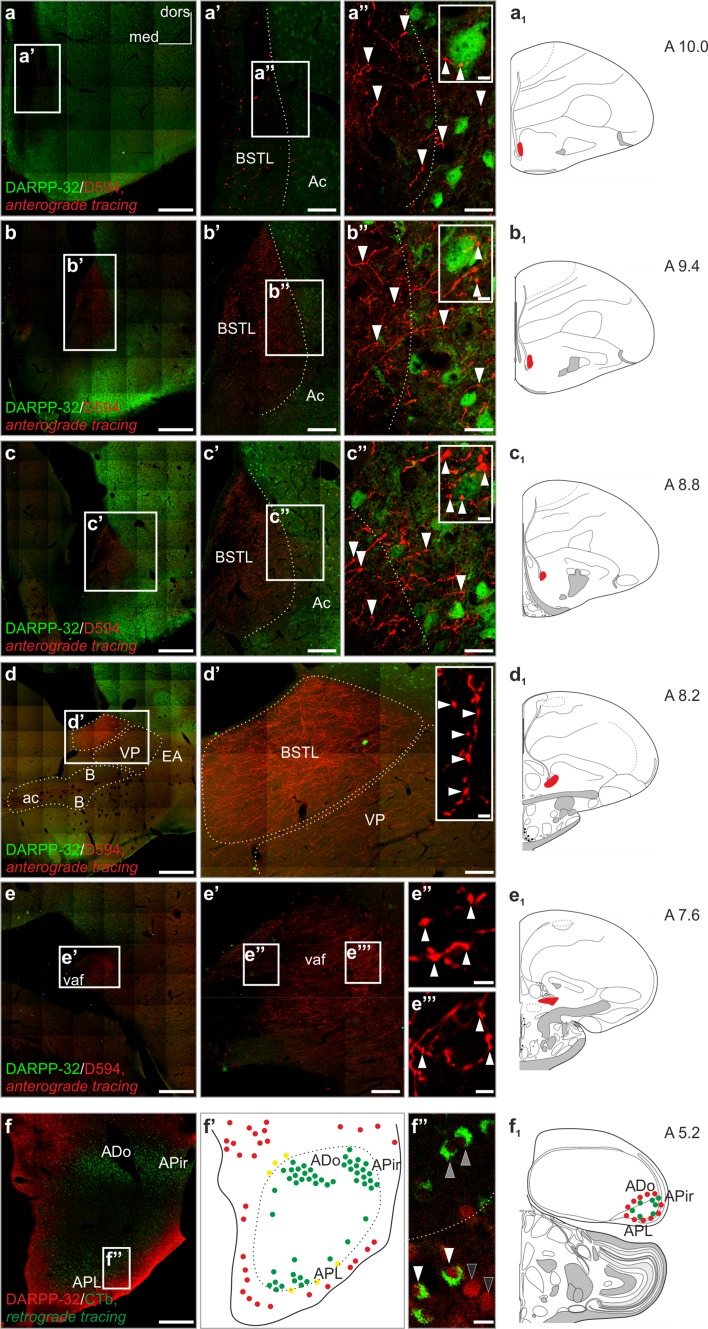
Immunolabelling against DARPP-32 combined with pathway tracing help to follow the path of arcopallial axons to their target regions within the ventrobasal forebrain. **a**–**e**_1_ Anterogradely labeled D594^+^ fibers pass through regions devoid of, but surrounded by DARPP-32^+^ cells. At different cranio-caudal levels (**a**, **a**_1_, **b**, **b**_1_, **c**, **c**_1_), D594^+^ axons terminate (**a**′, **b**′, **c**′) in two clearly separable regions: a dense terminal network in a DARPP-32 impoverished core region, largely corresponding to lateral part of the bed nucleus of stria terminalis (BSTL), and a less dense terminal network in the surrounding DARPP-32^+^ field, representing the nucleus accumbens (Ac) (*arrowheads* in **a**′′, **b**′′, **c**′′ indicate tracer-labeled axons). The *dotted line* in Figures **a**′–**a**′′, **b**′–**b**′′, **c**′–**c**′′ represents the putative border between the BSTL and Ac. At the level of the anterior commissure (ac) (**d**, **d**′, **d**_1_) where the Ac was no longer detectable, massive accumulation of D594^+^ fibers was visible (**d**′) in the BSTL, also extending to the adjacent subpallial regions: ventral pallidum (VP), extended amygdala (EA), and the basal nucleus of Meynert (B). The images **e**–**e**′′′ demonstrate the presence of anterogradely labeled axons in the ventral amygdalofugal tract (vaf, **e**–**e**′, axons in full focus in **e**′′ and **e**′′′). **f**, **f**_1_ Retrogradely labeled neurons in the arcopallium as traced with CTb from the Ac are surrounded by, but not intermingled with, DARPP-32^+^ neurons. In the diagram (**f**′), red circles label DARPP-32^+^ somata around the arcopallium (*black arrowheads* in **f**′′), *green circles* symbolize the distribution of retrogradely labeled perikarya (*gray arrowheads* in **f**′′). Occasionally, double-labeled cells (indicated by *yellow circles*, *white arrowheads* in **f**′′) also occur in the border zone. D594 Alexa Fluor^®^ 594 conjugated high-molecular-weight (10 kDa) dextran, *CTb* choleratoxin B subunit, *DARPP-32* dopamine- and cAMP-regulated phosphoprotein. *Scale bars* 1 mm (**a**–**f**), 50 µm (**a**′–**e**′), 20 µm (**a**′′–**c**′′, **f**′′), 5 µm (*insets* in **a**′′–**c**′′), 2 µm (**e**′′, **e**′′′)

Medial and lateral divisions of ventrobasal forebrain are characterized by the absence or presence of dopamine- and cAMP-regulated phosphoprotein containing (DARPP-32^+^) neurons, respectively, conferring potential functional differences. We hypothesized that the heterogeneity of this brain field might be reflected also by its arcopallial connectivity pattern. To test this, we investigated the ventrobasal juxtaventricular forebrain (including the BSTL and Ac) in serial coronal sections for anterogradely labeled fibers (D594-injection into the APir), and co-stained their target region for DARPP-32-immunoreactivity (Fig. 4). In the cranial sectional levels (A10–A8.8), the medial and lateral divisions of the ventrobasal forebrain could be clearly distinguished according to their DARPP-32-immunoreactivity (Fig. 4a–c′′). The majority of arcopallial axons ended in a medial DARPP-32 negative (DARPP-32^−^) subregion, corresponding to the BSTL (Fig. 4a′, a′′, b′, b′′, c′, c′′). However, although in lower densities, axons and terminal fields were also identified among the DARPP-32^+^ neurons in the adjacent lateral region, corresponding to the Ac (Fig. 4a′, a′′, b′, b′′, c′, c′′). In more caudal sectional levels (A8.2–A7.6), where the Ac has phased out, the axon terminals were found to be distributed in the BSTL (very massive amounts, d, d′) and also in the extended amygdala (EA), ventral pallidum (VP), and the nucleus basalis of Meynert (B), arriving via the vaf (e, e′′′).

Next, we investigated the relationship between DARPP-32-immunoreactivity and the source neurons of the arcopalliosubpallial tract. Using CTb as a retrograde tracer we identified labeled perikarya within the typical sites of origin in the arcopallium, which were surrounded by, but mostly did not coincide with, DARPP-32^+^ neurons (Fig. 4f–f_1_). Most of the arcopallium proved to show very weak or negative labeling agaist DARPP-32. In only a few cases did CTb^+^ neurons share DARPP-32-immunoreactivity (Fig. 4f′, f′′). Conclusively, we identified the amygdalosubpallial pathway which arises from mostly DARPP-32^−^ neurons to pass and terminate within largely DARPP-32^−^ regions, but with a select final target area also amongst DARPP-32^+^ neurons.

### Projection neurons of the arcopalliofugal (amygdalofugal) tract do not express the major neuronal calcium binding proteins parvalbumin, calbindin or calretinin

**Fig. 5:**
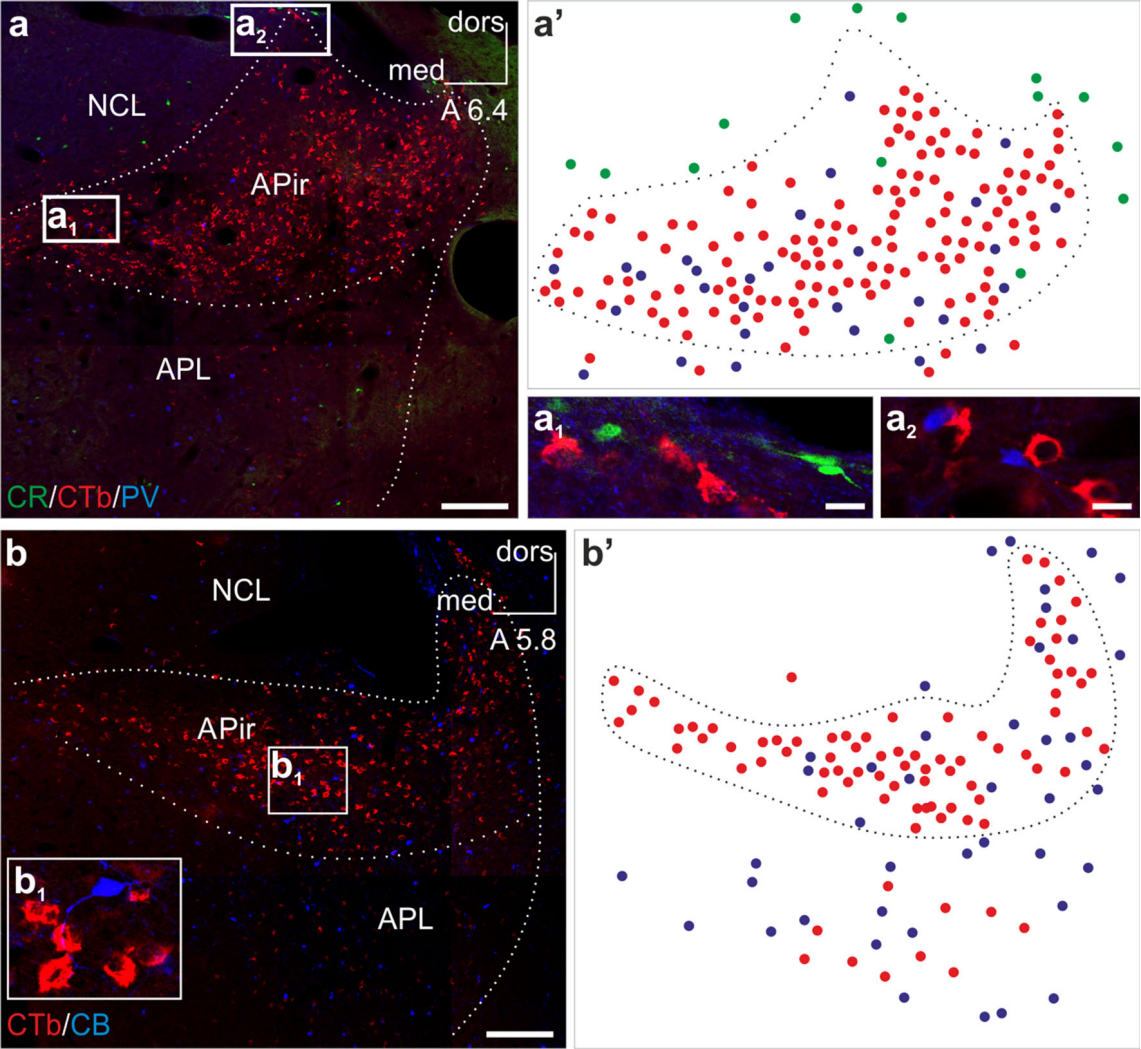
Arcopallial neurons projecting to BSTL and adjacent Ac are immunonegative for the major calcium binding proteins parvalbumin, calbindin and calretinin. (**a**, **a′**, **a**_**1**_, **a**_**2**_) The amygdalopiriform (APir) area of the arcopallium harbors a plethora of CTb^+^ neurons labeled retrogradely from the BSTL and adjacent Ac. These projection neurons do not express the calcium binding proteins calretinin or parvalbumin. Illustration (**a′**) shows the distribution pattern of single labeled calretinin^+^ (*green circles*), CTb^+^ (*red circles*) and parvalbumin^+^ (*blue circles*) neurons. (**b**, **b′**, **b**_**1**_) Similarly, retrogradely labeled CTb^+^ neurons remained immunonegative for the calcium binding protein calbindin in a more caudal part of the same arcopallial region. Illustration (**b′**) shows the distribution pattern of single labeled CTb^+^ (*red circles*) and calbindin^+^ (*blue circles*) neurons. (**a**, **b**) Cranio-caudal levels of the coronal sections are indicated as distance in millimeters AP according to Kuenzel and Masson (1988). *APL* posterolateral amygdala, *CB* calbindin, *CR* calretinin, *CTb* choleratoxin B subunit, *dors* dorsal, *med* medial, *NCL* caudolateral nidopallium, *PV* parvalbumin. *Scale bars* 200 µm (**a**, **b**), 10 µm (**a**_**1**_, **a**_**2**_)

We tested the possibility if arcopallial neurons projecting to the ventrobasal forebrain are distinct by the select expression of a calcium binding protein. In contrast to calretinin^+^ neurons which occurred, but only sporadically, in the chick arcopallium (Fig. 5a, a′), parvalbumin^+^ and calbindin^+^ neurons were detected throughout the arcopallium (Fig. 5a, a′, b, b′). Using retrograde tracing combined with multiple immunolabeling we showed that arcopalliofugal projection neurons did not express any of the major calcium binding proteins: CTb^+^ neurons remained invariably immunonegative for calretinin (Fig. 5a_1_), parvalbumin (Fig. 5a_2_) or calbindin (Fig. 5b_1_).

## Discussion

Retrograde tracing with choleratoxin B subunit (CTb), injected into the Ac, yielded labeled perikarya in a ring-shaped area of arcopallium, including the amygdalar dorsal region of Puelles et al. (2007) (ADo), corresponding to dorsal arcopallium (AD), according to Atoji et al. (2006); the amygdalohippocampal area (AHi) of Puelles et al. (2007), largely corresponding to the nucleus taeniae (TnA) of Atoji et al. (2006); the hilar amygdalar region (AHil) of Puelles et al. (2007), approximate correlate of medial arcopallium, parvocellular part (AMp), according to Atoji et al. (2006). A wedge-shaped node of dense accumulation of retrogradely labeled cells was observed in a laterodorsal subunit termed amygdalopiriform area (APir, Puelles et al. (2007)). The latter region largely coincided with the fields designated by Atoji et al. (2006) as the caudal ventrolateral nidopallium (NCVl), subnidopallium (SuN) and the posterior nucleus of arcopallium, compact division (PoAc), in the pigeon. Further retrogradely labeled cells were found in the posterolateral amygdala (APL) of Puelles et al. (2007), similar to the regions designated by Atoji et al. (2006) as basal posterior arcopallium (PoAb). Injections spreading into the BSTL led to similar distribution of labeled cells in the arcopallium. This is in agreement with the observation of Atoji et al. (2006) concerning the source region of BSTL projections. However, these authors restricted their analysis to the EA (including BSTL), not considering the Ac (corresponding to the generally accepted notion about the position of Ac back then). Later studies (Balint and Csillag 2007; Balint et al. 2011; Husband and Shimizu 2011) have led to a reappraisal of the position of Ac subregions in the domestic chicken (in the vicinity of BSTL throughout the rostrocaudal extent A8.8–A10.6 of ventrobasal forebrain, according to the coordinates of Kuenzel and Masson (1988)). Thus, overlapping simultaneous projections from the arcopallium to both BSTL and Ac have become a distinct possibility.

Notably, the ATn was largely devoid of retrogradely labeled neurons unlike the adjacent AHil region, which contained abundant CTb^+^ cells. In addition to arcopallial sources, retrogradely labeled neurons were also seen in the EA, particularly its border region with the arcopallium.

The position of source neurons for the arcopallial-accumbens pathway was verified also by anterograde pathway tracing. The results of more refined analysis, based on discrete subregional injections, show that the fibers arising from the APir are likely to reach the Ac (in addition to BSTL), whereas those arising from the dorsal and medial arcopallial subdivisions mainly innervate the BSTL and EA only. Overall, the projection to any ventrobasal target area was more dense in those cases, where the tracer had been deposited in the laterodorsal (APir) area of arcopallium, in agreement with a greater density of source neurons there (as detected by retrograde tracing).

The study enabled precise topographic description of the course of the arcopalliofugal pathway (essentially corresponding to the amydalofugal pathway in question). It derives from two main output fiber streams, also mentioned by Atoji et al. (2006): one along the dorsal border of arcopallium, presumably corresponding to the stria terminalis of mammals, and another ventral tract along the ventral pallial border (putative equivalent of the ansa peduncularis of mammals). Further course of the pathway can be traced in our material as follows. The fibers arising from caudal levels follow a dorsal course and enter the vaf. Then, having bypassed the ATn, they traverse the subpallial (extended) amygdala (with profuse terminal fields), and the BSTL (also terminating there in large numbers) before invading the shell and the core of Ac. The fibers arising from levels that are more rostral mainly follow a ventral course, passing through the ventrobasal part of EA, and then invading the nucleus basalis and olfactory tubercle. Efferents were also observed in the ventral pallidum, lateral septum and diagonal band. It has to be noted that the nucleus taeniae (ATn) of Puelles et al. (2007), adjacent to the vaf, is not identical with the nucleus termed TnA by Atoji et al. (2006), which is placed at some distance from the vaf. We reconstructed the course of the amygdalofugal pathway in a pseudo-3D (movie) format (Electronic Supplementary Material 2).

The presence of DARPP-32 has been well established, also in avian brain regions (Durstewitz et al. 1998; Roberts et al. 2002). This protein is an important signaling molecule present in dopaminoceptive neurons (Hemmings et al. 1987). In agreement with previous observations (Schnabel et al. 1997), in our study DARPP-32 immunoreactivity was present in all striatal regions (including the Ac), but the BSTL was largely devoid of DARPP-32. DARPP-32 in ‘NST’ (an earlier name variant for BSTL) has been reported poor staining by Reiner et al. (1998), together with a low density of substance P (SP). DARPP-32 labeling was found to be prominently weaker in the BSTL than in the surrounding ventral striatum (identified as the rostral pole of Ac) (Balint and Csillag 2007). Thus, immunoreactivity for DARPP-32 could be used as a marker distinguishing adjacent Ac and EA regions. Interestingly, Ac-bound arcopallial neurons were devoid of DARPP-32, except for a few cells in the lateral nidopallium bordering the dorsal arcopallium. Apparently, a ring of DARPP-32 containing cells surrounds the arcopallial source region of the amygdalofugal pathway, without considerable overlap. Massive labeling against DARPP-32 in the caudolateral nidopallium and piriform cortex, adjacent to the APir (but not in central arcopallial fields) has been observed also by Schnabel et al. (1997). In the same study, the amount of TH labeling was found to be very high in the dorsal and laterodorsal arcopallium (overlapping the source regions of our present study), which otherwise showed weaker labeling to DARPP-32. In most cases, absence of this signaling molecule does not involve a similar lack of dopaminergic innervation. DARPP-32 labeling was found to be low in the ‘Ac’, despite a dense staining of TH fibers (Schnabel et al. 1997). It has to be noted that the region defined by these authors as Ac was later renamed BSTL (Reiner et al. 2004). Distribution of DARPP-32 labeling, if overall similar, was by no means an exact match of the distribution of dopamin D1 receptors (Ball et al. 1995), in the quail (Absil et al. 2001).

An important finding is that the source cells of the amygdalofugal tract specified in the present study are devoid of calbindin, calretinin and parvalbumin, albeit these calcium-binding proteins do occur in many neighboring cells, profusely intermingling with the retrogradely traced neurons. All three calcium-binding proteins are known to be widely distributed in various subregions of mammalian amygdala (Pitkanen and Kemppainen 2002). The presence of calcium binding proteins has been typically exploited for the identification of functionally distinct neuronal subsets also in the avian brain (Gati et al. 2014; Husband and Shimizu 2011; Pfeiffer and Britto 1997; Roberts et al. 2002; Suarez et al. 2006). The arcopallium harbors subsets of parvalbumin^+^, calbindin^+^ and calretinin^+^ neurons (Cornez et al. 2015; Roberts et al. 2002). The observed lack of calcium binding proteins is in harmony with our previous finding that at least a contingent of the source neurons of the amygdalofugal pathway are excitatory based on the presence of glutamate and aspartate in asymmetrical synaptic terminals (of excitatory morphological type), deriving from amygdalofugal axons (Hanics et al. 2012). The calcium binding proteins calbindin D28K, calretinin and parvalbumin tend to occur in smooth non-pyramidal interneurons (and some pyramidal neurons) of mammalian cortex (for review: DeFelipe (1997)). At least in cortical fields, these calcium-binding proteins mark specific classes of inhibitory interneurons (Hof et al. 1999).

Of the arcopallial subregions yielding the densest projections to BSTL and Ac, the dorsolateral arcopallium and neighboring caudal nidopallial regions have been considered to be lateral pallial derivatives and homologous to the basolateral amygdala of mammals or reptiles (Guirado et al. 2000; Lanuza et al. 1998; Martinez-Garcia et al. 2002, 2008; Redies et al. 2001). However, this has been disputed by other authors, categorizing the regions rather as ventral pallial derivatives (Medina et al. 2004; Puelles et al. 2000), though maintaining the possibility of part of basolateral amygdala being ventral pallial (Abellan et al. 2009). Thus, the main source region for projections directed to BSTL/Ac may still be categorized, as equivalent of mammalian BLA, since a lateral pallial origin, at least in part, has not been ruled out. This interpretation is in agreement with Moreno and Gonzalez (2006), placing the APir and caudolateral nidopallium (NCL) into a lateral pallial zone, while other, less dense source regions (ADo, AHi, AHil) would already belong in the ventral pallial field. The ATn (amygdaloid taenial nucleus, designated as pallial medial amygdalar nucleus, PMA, by Abellan et al. (2009) was devoid of retrogradely labeled cells, at least at the sectional levels of its largest extension. This region did not contain anterogradely labeled fibers either; the fibers seem to pass by the nucleus without termination. In addition to pallial amygdalar sources of the pathway, retrogradely labeled cells were also observed en route in the extended amygdala, especially in the region adjacent to the arcopallium (including the capsular central amygdala, intercalated cell patches, peri-INP island field and the oval central amygdalar nucleus, according to the categories by Vicario et al. (2014).

Convergent amygdalar input to the EA and BSTL, as well as to Ac subregions likely transmits contextual fear and aggression-related signals to both viscerolimbic (EA) and learned reward- and motivation-related (Ac) ventrobasal forebrain regions. Fear responses have been attributed to either the central amygdala or the pallial laterobasal amygdala (Davis and Whalen 2001). According to previous observations, the BSTL is primarily involved in contextual fear (Duvarci et al. 2009; Phelps and LeDoux 2005; Walker and Davis 2008), whereas the central amygdala is more involved in lasting fear responses, similar to anxiety (Duvarci et al. 2009; Walker and Davis 2008; Walker et al. 2003, 2009). Based on evidence from the previous (Hanics et al. 2012) and present observations, this pathway is excitatory, with potential cotransmission of Glu and Asp. Dopaminergic input to the source neurons of this pathway is unlikely to involve DARPP-32 as the main signal transducer, as evidenced by the present study.

There appears to be a certain degree of overlap between the numerous subregions of EA and those of the Ac (in particular the shell). The EA can be envisaged as a network of neurons of multiple origins, extending from selected nuclei of the amygdala to specific areas of the ventrobasal forebrain. In the course of development, these neurons originating from subpallial (medial ganglionic eminence, lateral ganglionic eminence, preoptic) and pallial (esp. ventral pallial) primordia followed specific migratory routes or cell subcorridors (Vicario et al. 2015), e.g., the stria terminalis. Because of migration, cellular clusters originating from one domain may invade the territories of other domains, rendering the borders ‘fuzzy’. This may well be the case with the border between the Ac and BSTL or, even more so, the border between Ac and rostral extended amygdala (SpAr). Yet certain cellular characteristics may be preserved in spite of overlapping migration. For example, a recently described calcium binding protein, secretagogin, known to occur in EA regions of mammals (Mulder et al. 2010) labels clusters of selected neurons also in the subpallial amygdala, including BSTL, of domestic chickens, whereas the Ac (together with the striatal complex) are impoverished in secretagogin label (Gati et al. 2014). Based on a detailed study on specific transcription factors and cell tracking in chicken (Vicario et al. 2015), the BSTLd (as defined in the paper) contains neurons of both pallidal and striatal origin. While BSTL develops in the pallidal domain of the forebrain, Ac is a ventral striatal derivative. According to Vicario et al. (2015), the amount of Islet1 (taken as a marker for the ventral striatal domain) was significant, mainly in the dorsomedial BSTL but also visible in the dorsolateral subregion, interspersed with Pax6 and Nkx.2.1., pointing to a potential ‘overflow’ from Ac. The area defined SpAr may also encroach upon territories of Ac shell [Alheid et al. 1995; de Olmos et al. 2004), see further discussion of the question by Alba Vicario, doctoral thesis (2015)].

Of the two known subregions of EA (central and medial) (Martinez-Garcia et al. 2008), the central EA includes the BSTL, and is implicated in fear and aggression-related behaviors. Thus, the BSTL, relevant central amygdalar components and specific neural groups, scattered along the path of the stria terminalis and the associated vaf, are likely involved in mediating these modalities to other viscerolimbic centers (diagonal band nucleus, septum, and hypothalamus and forebrain cholinergic system). However, the very same information is salient also to the processing of reward, aversion and memory formation for these modalities, as well as the initiation of locomotor response and cognitive functions based thereupon, all considered to be typical for the Ac. This dichotomy of viscerolimbic-related and reward-related amygdalar input may be represented in the described pathway of the domestic chicken, terminating in both EA and Ac regions.

## Electronic supplementary material

Below is the link to the electronic supplementary material.

Supplementary material 1 (PDF 2384 kb)

Supplementary material 2 (PDF 7 kb)

Supplementary material 3 (AVI 93608 kb)

## Abbreviations

Ac: Nucleus accumbens
ac: Anterior commissure
ACo: Core of the arcopallium
AD: Dorsal arcopallium
ADo: Dorsal part of the acopallium/amygdalar dorsal region
AHi: The amygdalohippocampal area
AHil: Hilar part of the arcopallium/hilar amygdalar region
Amp: Medial arcopallium, parvocellular part
APir: Amygdalopiriform area
APL: Posterolateral part of the arcopallium/posterolateral amygdala
Asp: l-Aspartate
ATn: Taenial part of the arcopallium/amygdaloid taenial nucleus
B: Nucleus basalis of Meynert
BSTL: Bed nucleus of stria terminalis, lateral part
CB: Calbindin
CR: Calretinin
CTb: Choleratoxin B subunit
D594: Alexa Fluor^®^ 594 conjugated high-molecular weight (10 kDa) dextran
DARPP-32: Dopamine- and cAMP-regulated phosphoprotein
DLM: Medial part of the dorsolateral anterior thalamic nucleus
DMA: Anterior dorsomedial nucleus (thalamus)
EA: Extended amygdala
FB: Fast blue
Glu: l-glutamate
MSt: Medial striatum
NCVl: Caudal ventrolateral nidopallium
PMA: Pallial medial amygdalar nucleus
PoAb: Basal posterior arcopallium
PoAc: Posterior nucleus of arcopallium, compact division
PV: Parvalbumin
SpAr: Rostral subpallial (extended) amygdala
SuN: Subnidopallium
TH: Tyrosine hydroxylase
TnA: Nucleus taeniae
vaf: Ventral amygdalofugal pathway
VP: Ventral pallidum
