# Supplementary material for: Multiple amygdaloid divisions of arcopallium send convergent projections to the nucleus accumbens and neighboring subpallial amygdala regions in the domestic chicken: a selective pathway tracing and reconstruction study
Source: Brain Struct Funct. 2016 Apr 6;222(1):301–15. doi: 10.1007/s00429-016-1219-8 (PMC5225175; doi:10.1007/s00429-016-1219-8)
Supplement: Supplementary file 1 — Supplementary material 1 (PDF 2384 kb) [file 429_2016_1219_MOESM1_ESM.pdf]

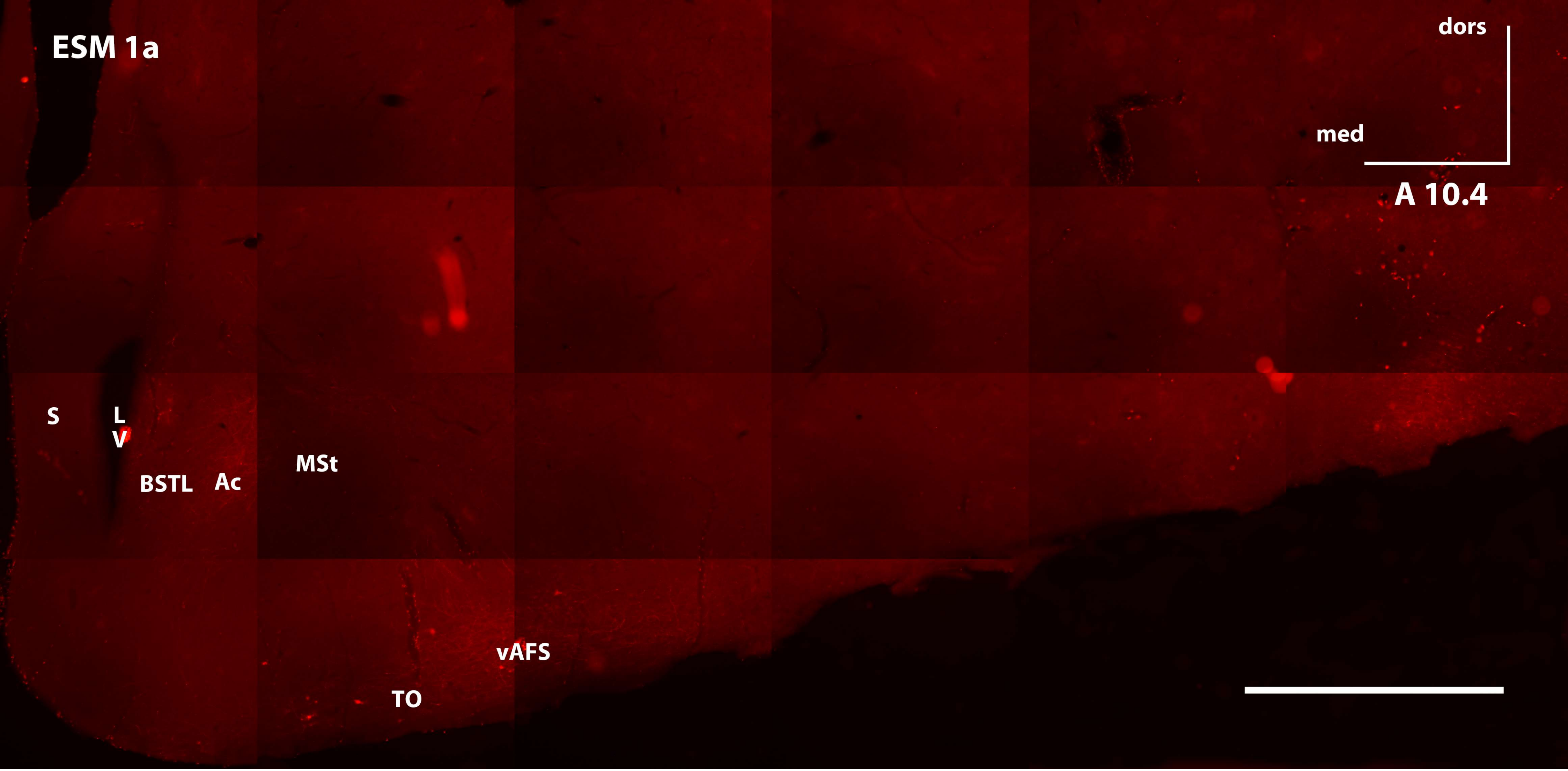

Hanics et al. 2015 Brain Structure and Function

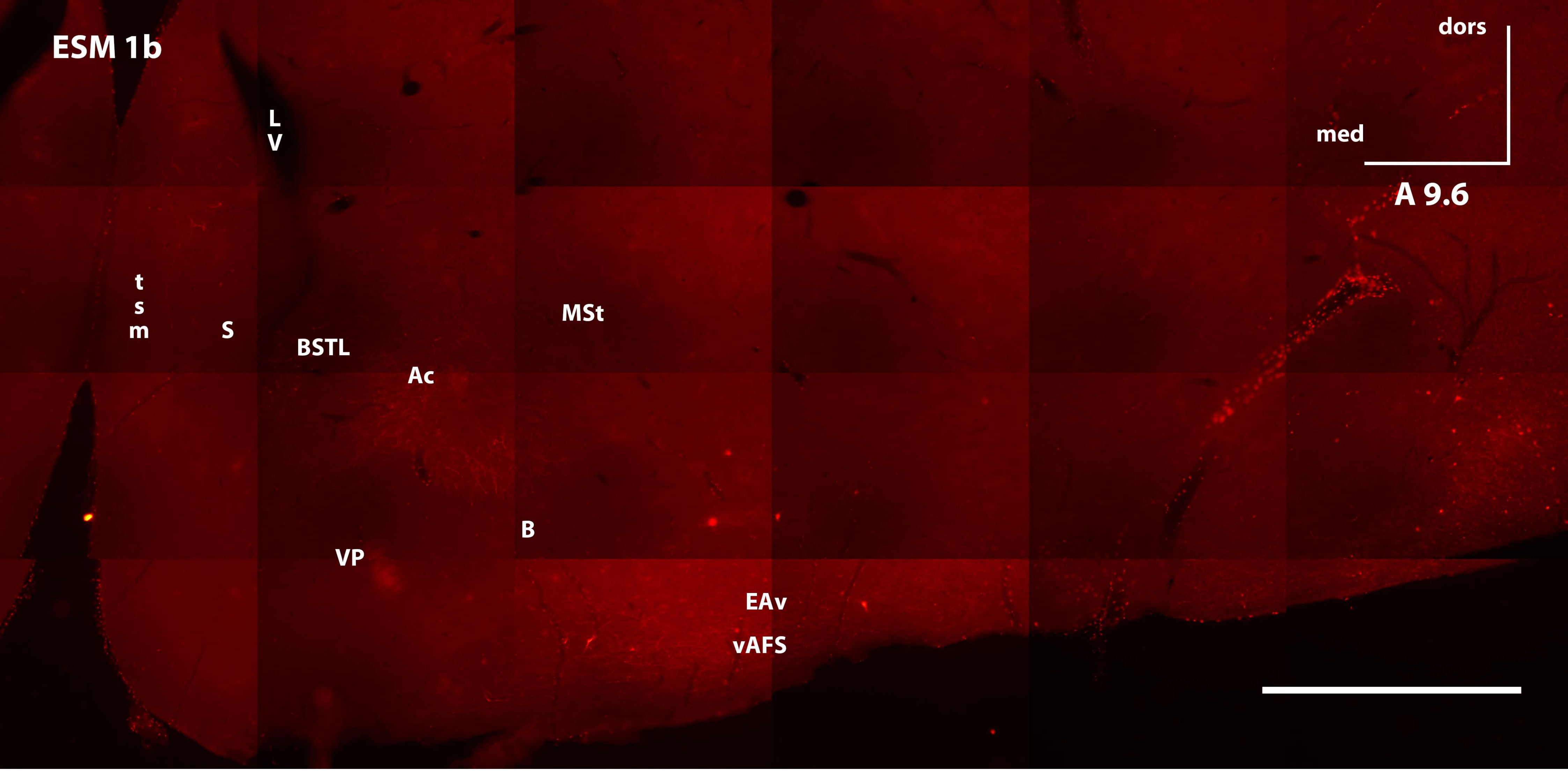

Hanics et al. 2015 Brain Structure and Function

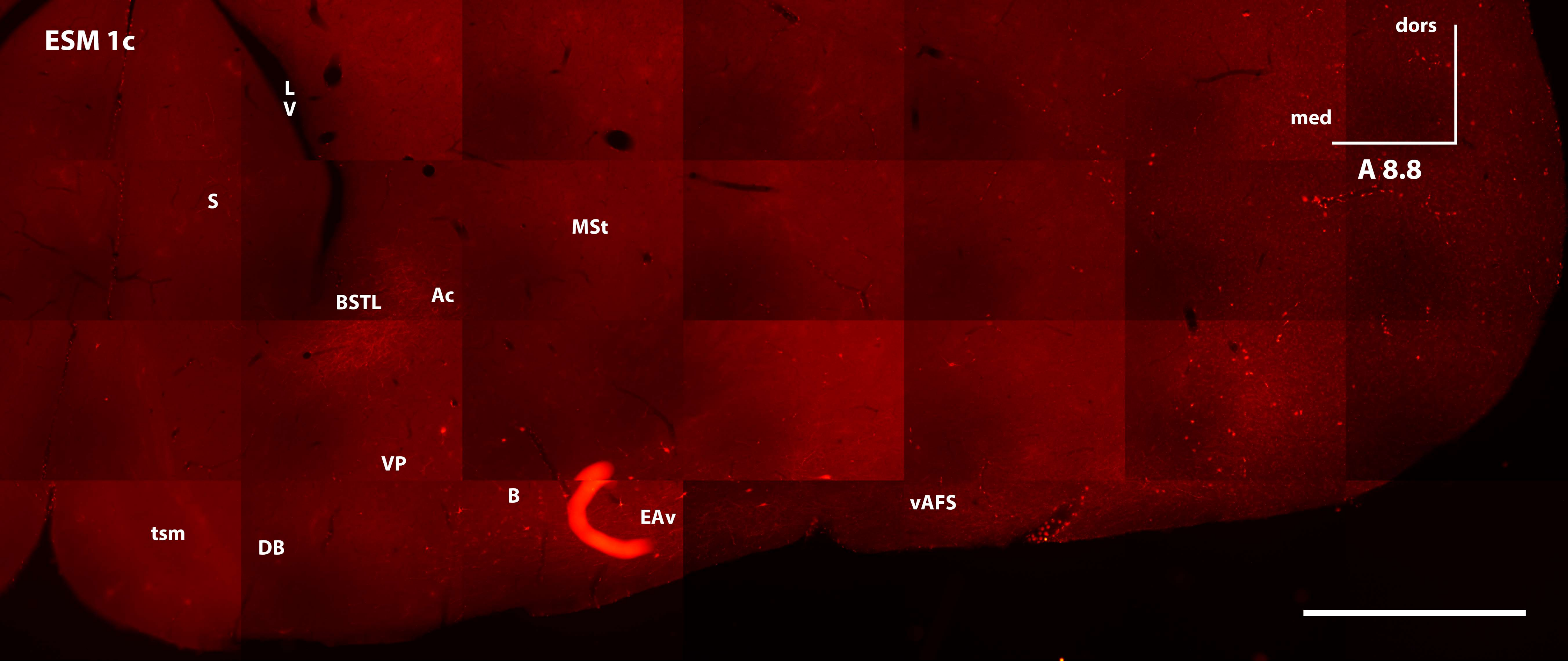

Hanics et al. 2015 Brain Structure and Function

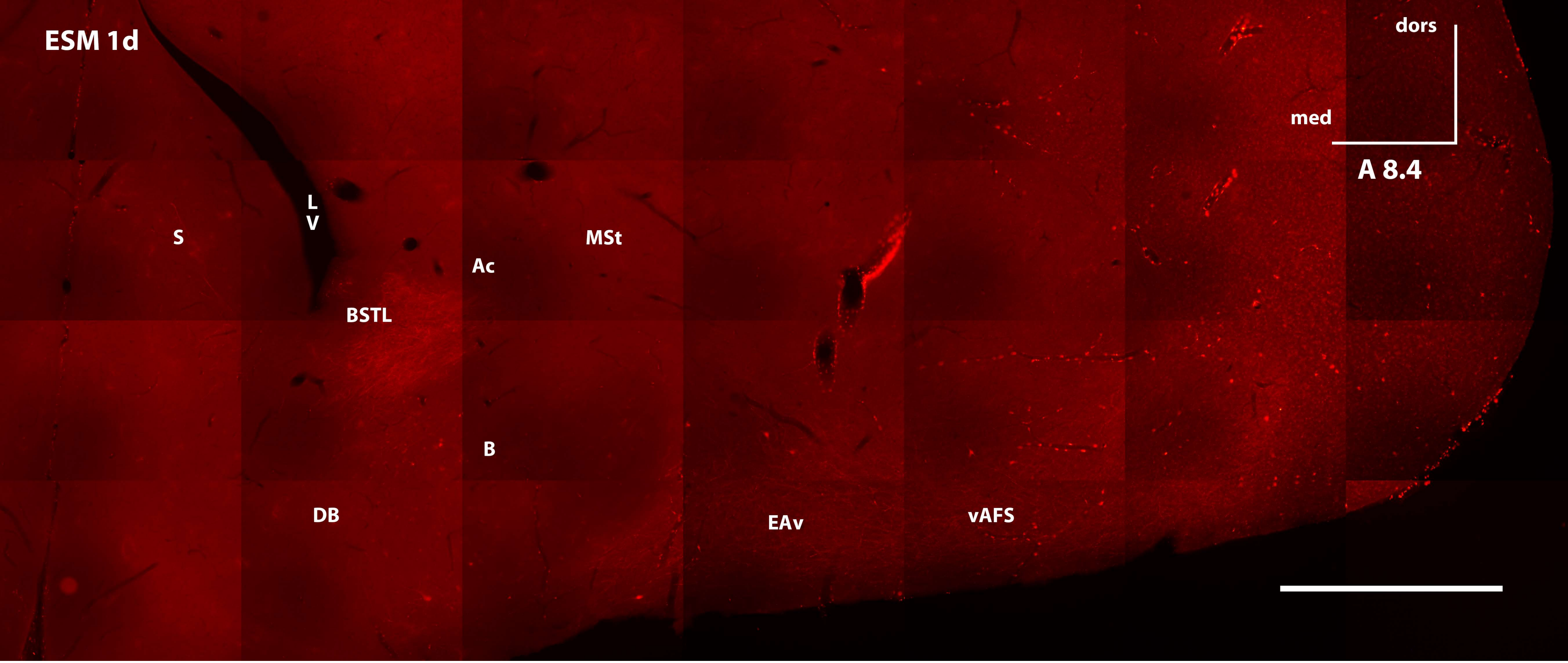

Hanics et al. 2015 Brain Structure and Function

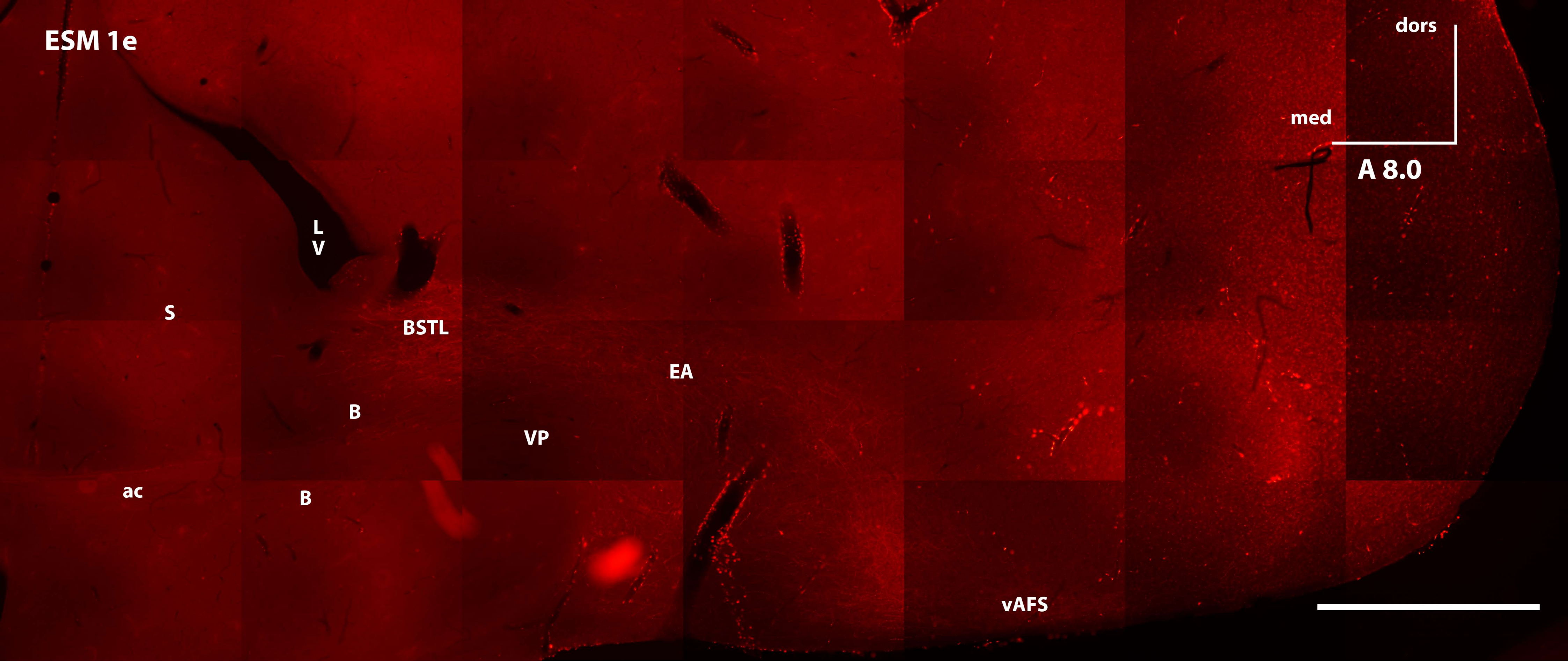

Hanics et al. 2015 Brain Structure and Function

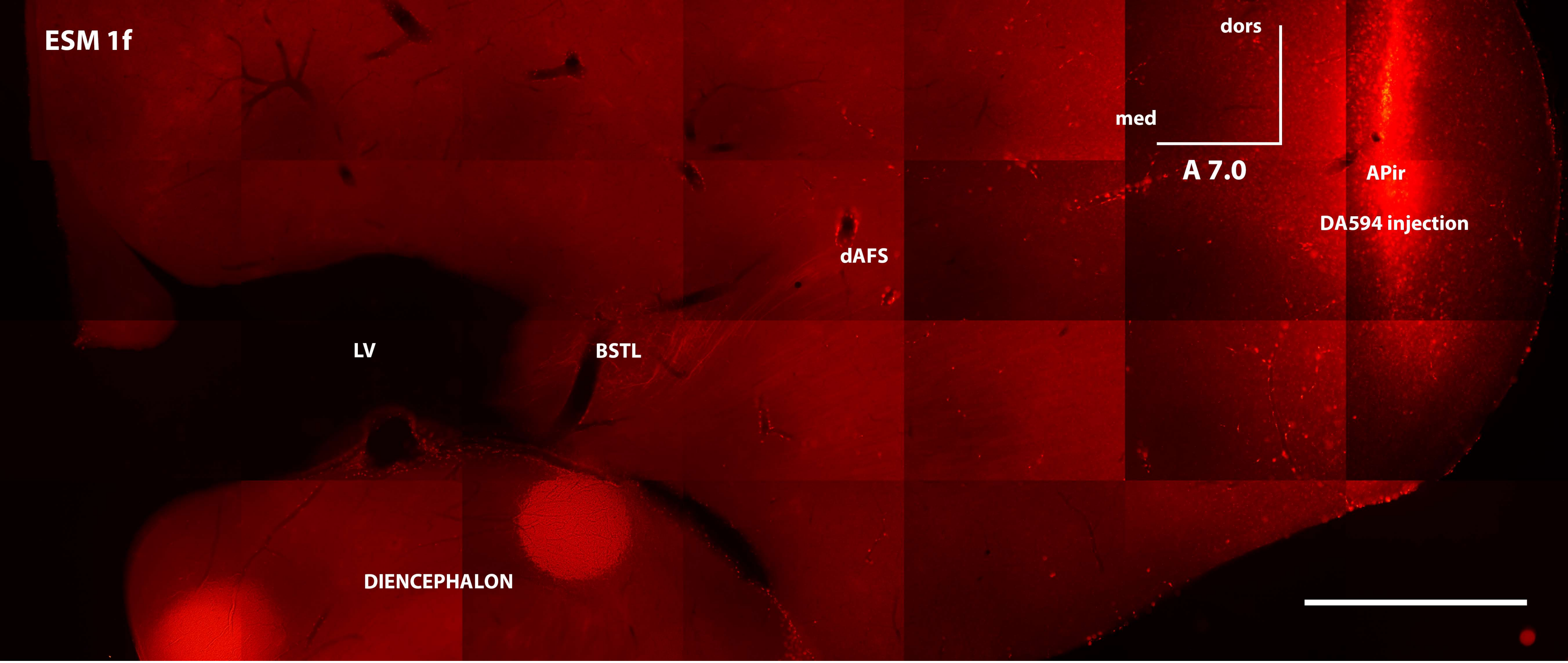

Hanics et al. 2015 Brain Structure and Function

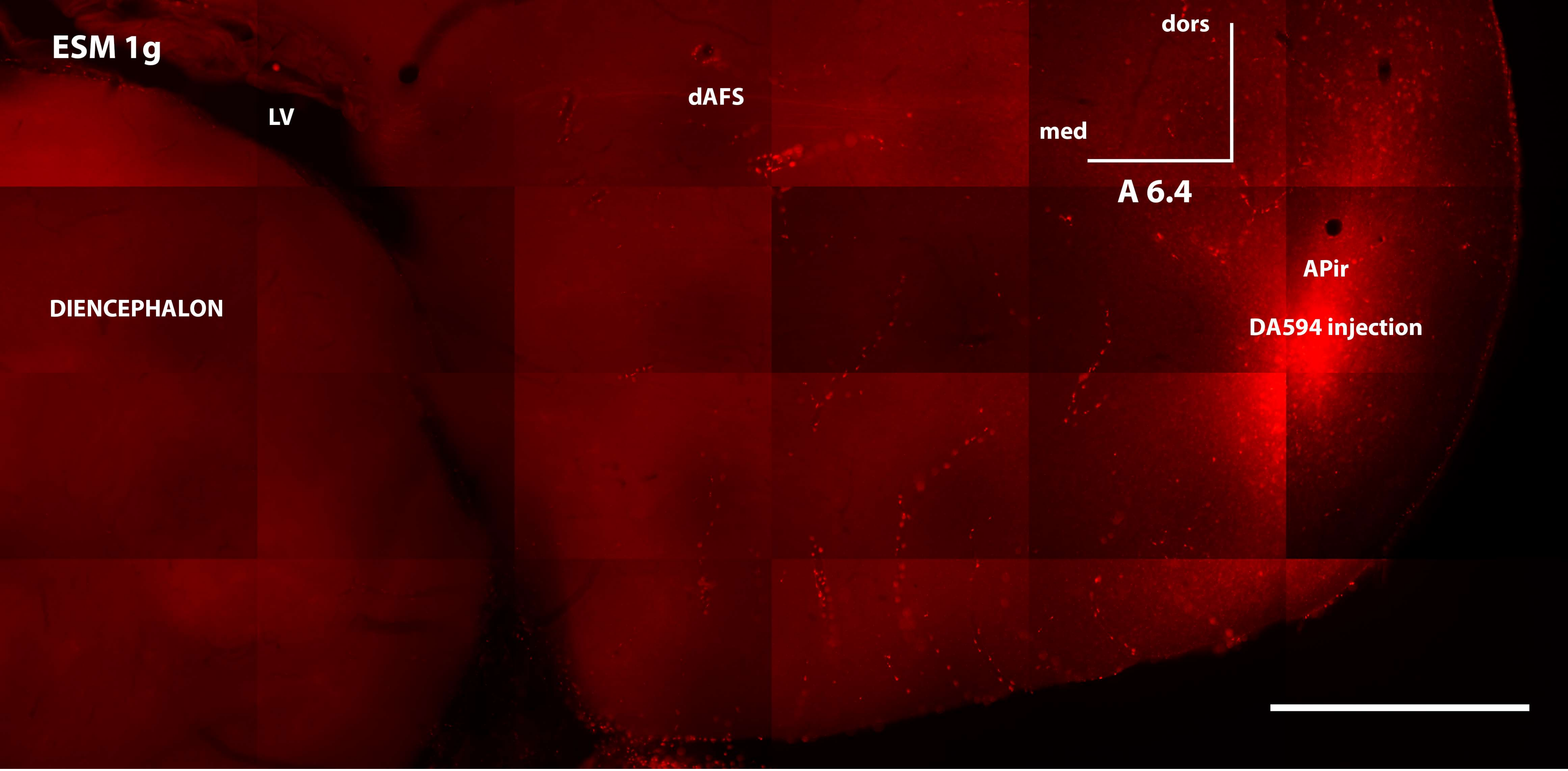
ESM 1g
LV
dAFS
dors
med
A 6.4
APir
DA594 injection
DIENCEPHALON
