# Supplementary material for: Multiple amygdaloid divisions of arcopallium send convergent projections to the nucleus accumbens and neighboring subpallial amygdala regions in the domestic chicken: a selective pathway tracing and reconstruction study
Source: Brain Struct Funct. 2016 Apr 6;222(1):301–15. doi: 10.1007/s00429-016-1219-8 (PMC5225175; doi:10.1007/s00429-016-1219-8)
Supplement: Supplementary file 2 — Supplementary material 2 (PDF 7 kb) [file 429_2016_1219_MOESM2_ESM.pdf]

Hanics et al. 2015, Brain Structure and Function

Electronic Supplementary Material 1a-g

Multi-tile composite images of brain sections following D594 anterograde tracer injection into the APir. The course of labeled fibers can be followed between A10.4 to A6.4. Cranio-caudal levels of the coronal sections are indicated as distance in millimeters according to Kuenzel and Masson (1988). Scale bar: 1mm *Abbreviations: ac* anterior commissure, *Ac* nucleus accumbens, *APir* amygdalopiriform area, *B* Basal nucleus (Meynert), *BSTL* lateral part of the bed nucleus of stria terminalis, *DB* nucleus of diagonal band, *D594* dextran (10kDa), *EA* extended amygdala, *EA*v extended amygdala ventral part, *LV* lateral ventricle, *MSt* medial striatum, *S* septum, *tsm* tractus septomesencephalicus, *dAFS* dorsal amygdalofugal output fiber stream, *vAFS* ventral amygdalofugal output fiber stream, *VP* ventral pallidum, *TO* tuberculum olfactorium, *dors* dorsal, *med* medial

Electronic Supplementary Material 2

Moving picture (.avi) reconstruction of the arcopalliofugal pathways, generated from two section series containing the dorsal and ventral tracts, detected by anterograde tracing with D594. Color coding: *Brain contours* turquoise, *ventricular system* yellow, *BSTL* green, *arcopallium* magenta, *dorsal amygdalofugal output fiber stream* red, *ventral amygdalofugal output fiber stream* blue
